# Supplementary material for: Circulating exosomal long noncoding RNA PRINS—First findings in monoclonal gammopathies
Source: Hematol Oncol. 2018 Sep 13;36(5):786–91. doi: 10.1002/hon.2554 (PMC6585732; doi:10.1002/hon.2554)
Supplement: Supplementary file 1 — Supplementary Figure S1: Correlation of translocation t(4;14) with exosomal PRINS levels in MM patients Supplementary Table S1: Correlation of serum lncRNA with biochemical parameters. For correlation of the data, Spearman coefficient was adopted; significant coefficients of correlation (P < 0.05) are marked with bold and italics. [file HON-36-786-s001.docx]

**Circulating exosomal long non-coding RNA PRINS – first findings in monoclonal gammopathies**

**Running head:** Exosomal lncRNA PRINS as a marker of monoclonal gammopathies

Lenka Sedlarikova^1,¶^, Bozena Bollova^1,¶^, Lenka Radova^2^, Lucie Brozova^3^, Jiri Jarkovsky^3^, Martina Almasi^4^, Miroslav Penka^4^, Petr Kuglík^5^, Viera Sandecká^6^, Martin Stork^6^, Ludek Pour^6^, Sabina Sevcikova^1,4,*^

^1^ Babak Myeloma Group, Department of Pathological Physiology, Faculty of Medicine, Masaryk University, Brno, Czech Republic

^2^ Central European Institute of Technology, Masaryk University, Brno, Czech Republic

^3^ Institute of Biostatistics and Analyses, Faculty of Medicine, Masaryk University, Brno, Czech Republic

^4^ Department of Clinical Hematology, University Hospital Brno, Brno, Czech Republic

^5^ Department of Experimental Biology, Faculty of Science, Masaryk University, Brno, Czech Republic

^6^ Department of Internal Medicine, Hematology and Oncology, University Hospital Brno, Brno, Czech Republic

*Corresponding author:

Sabina Sevcikova

Babak Myeloma Group

Department of Pathological Physiology

Faculty of Medicine, Masaryk University

Kamenice 5, Brno 625 00

Czech Republic

[sevcik@med.muni.cz](mailto:sevcik@med.muni.cz)

tel. no: + 420 604 864 989

fax no: + 420 5 4949 4340

^¶^ These authors contributed equally to this work.

**Keywords:** multiple myeloma, qPCR, long non-coding RNA, monoclonal gammopathy of undetermined significance, biomarker

Manuscript word count: 2534

This work was supported by grant of the Czech Ministry of Health AZV 15-29508A.

**Abstract**

Multiple myeloma is the second most common hematological malignancy characterized by focal lesions of malignant plasma cells in the bone marrow. These lesions contain subclones that directly influence survival of patients. Bone marrow biopsies are single-site biopsies and thus cannot contain all information about the tumor. In contrast, liquid biopsies analyze circulating cells and molecules that are secreted from all sites of the tumor. Long non-coding RNA molecules are one class of these molecules. We performed a two-phase biomarker study investigating lncRNA expression profiles in exosomes of peripheral blood serum of newly diagnosed multiple myeloma (MM) patients, monoclonal gammopathy of undetermined significance (MGUS) patients in comparison to healthy donors (HD). Surprisingly, this analysis revealed dysregulation of only one exosomal lncRNA PRINS in MM vs. HD. Overall, MM and MGUS patients were distinguished from HD with sensitivity of 84.9% and specificity of 83.3%. Our study suggests a possible diagnostic role for exosomal lncRNA PRINS in monoclonal gammopathies patients.

**Introduction**

Monoclonal gammopathies (MG), including multiple myeloma (MM) and monoclonal gammopathy of undetermined significance (MGUS), are diseases characterized by malignant proliferation of clonal plasma cells in the bone marrow (BM) (1).

MM is a heterogeneous disease with focal lesions in the BM but also elsewhere in the body; these lesions contain subclones that directly influence survival of patients as well as response to treatment (1). Therefore, analysis of biopsy specimen obtained from a single site in the BM does not contain information about all pathological clones (2). Liquid biopsies (biopsies of peripheral blood) represent a real promise for such diseases since circulating molecules detectable in peripheral blood (PB) mirror the complex heterogeneity of MG and can serve as potential diagnostic, prognostic and predictive markers. We and others showed that these molecules include cell-free DNA (2, 3) and non-coding RNA (ncRNA), especially microRNA (miRNA) (4 - 6) and long non-coding RNA (lncRNA) (7). LncRNA expression is tissue-specific and implicated in diverse biological functions (8). LncRNA are involved in tumorigenesis but also tumor progression and metastases (9). These molecules also circulate in body fluids (2, 7). This two-phased biomarker study focused on circulating lncRNA as potential diagnostic markers of MG.

**Materials and Methods**

**Patients and healthy donors**

In total, 141 serum samples obtained from newly diagnosed MM patients (56 samples), MGUS patients (49 samples) and HD (36 samples) were evaluated for this study (Table 1). MM and MGUS patients were diagnosed according to the International Myeloma Working Group (IMWG) guidelines (1). All patients’ samples were collected at the time of diagnosis prior to treatment. HD samples were age/sex matched to patients as described in Table 1. All patients were diagnosed at the University Hospital Brno, Czech Republic, and signed the informed consent form approved by the Ethics committee of the hospital in accordance with the current version of the Helsinki Declaration.

**Sample preparation and exosome isolation**

Serum samples were collected as previously described (4). Serum exosomes were isolated by miRCURY Exosome Isolation Kit (Exiqon, Vedbæk, Denmark) according to manufacturer’s protocol and characterized by transmission electron microscopy (negative dyeing, 2% ammonium molybdate). Exosomal fraction of serum was used for total RNA extraction as described below.

**Extraction of total RNA**

Total RNA was extracted using miRNeasy Serum/Plasma Kit (Qiagen, Hilden, Germany) based on manufacturer’s instructions. RNA quantity and purity was determined spectrophotometrically using NanoDrop ND-1000 Spectrophotometer (Thermo Scientific, Wilmington, DE, USA).

**Screening phase of the study - lncRNA profiling**

In total, 84 candidate lncRNA were determined by RT2 lncRNA PCR Array – Human lncRNA Finder (Qiagen, Germany) using exosomal fraction of serum of 6 MM patients and 6 HD in the screening part of the study according to manufacturer’s instructions on 7500 Real-Time PCR System. Analysis of data was performed using SDS version 2.0.1 software (Applied Biosystem, USA). The relative expression levels of target lncRNA were determined as 2^−ΔCt^. Average values of three most stable reference genes – *B2M*, *RPLP0* and *RN7SK* - were used for normalization.

**Validation of candidate lncRNA by qPCR**

For validation, significantly deregulated lncRNA from the screening phase (PRINS, LINC‑ROR) and previously published lncRNA (UCA1, NEAT1) (10) were used. These candidate lncRNA were validated using the relative quantification approach 2^-ΔCt^ on 50 MM patients, 49 MGUS patients and 30 HD. After RNA extraction, High-capacity cDNA reverse transcription kit (Applied Biosystems, USA) was used according to manufacturer’s recommendations. Expression levels of LINC‑ROR, PRINS, UCA1 and NEAT1 were detected by RT-qPCR using TaqMan non-coding RNA assays (UCA1: Hs01909129_s1, NEAT1: Hs03453535_s1), TaqMan Gene Expression Assays (LINC‑ROR: Hs04332550_m1, PRINS: Hs03671803_s1) (all Applied Biosystems, USA). Ct values were obtained using 7500 SDS Software v 1.4.0 (Applied Biosystems, USA). Relative expression levels of target lncRNA were determined by the equation 2^-ΔCt^. The *18S rRNA* reference gene was selected by comparing the expression levels of twelve candidate reference genes from Reference Gene Panel Human (TATAA Biocenter, Sweden). The *18S rRNA* gene was chosen as reference gene.

**Interphase fluorescence *in situ* hybridization analysis (I-FISH)**

I-FISH was performed as described previously (11) and examined for presence of gain(1)(q21), del(13)(q14), del(17)(p13), t(4;14) and hyperdiploidy status of MM patients. Available I-FISH data are listed in Table 1.

**Statistical evaluation**

Expression data from lncRNA profiling were statistically evaluated in the environment of statistical language R by use of Bioconductor package and LIMMA approach. P-values below 0.05 were considered as statistically significant. P-values from profiling were adjusted according to Bonferroni correction for multiple comparisons.

Standard descriptive statistics were applied. Statistical significance of differences in continuous variables among groups of patients was analyzed using nonparametric Kruskal‑Wallis or Mann-Whitney U test. For the robust analysis of continuous parameters relationship, the Spearman correlation coefficient was used.

Receiver Operating Characteristic (ROC) was used to determine sensitivity and specificity of each lncRNA or their combinations based on multivariate logistic regression model and area under the curve (AUC). Survival rates were estimated using the Kaplan-Meier method. Univariate and multivariate Cox proportional hazards models were used to assess the association of prognostic factors with overall survival. The variables in the multivariate model were chosen according to clinical significance in MM patients’ evaluation and survival prediction. Statistical analysis was performed using the GraphPad Prism 5 (GraphPad Software, San Diego, CA, USA), MedCalc Statistical Software v.14.8.1 (MedCalc Software, Ostend, Belgium) and IBM SPSS Statistics for Windows, Version 23.0 released in 2013 (IBM Corp., Armonk, NY, USA).

**Results**

**Screening phase of the study - lncRNA profiling**

In the screening phase of the study, we determined expression profiles of 84 lncRNA in serum exosomes of 6 MM patients *vs*. 6 HD. We identified lncRNA PRINS as differentially expressed in MM patients (all adjusted p < 0.042) (Figure 1). We selected UCA1, NEAT1, LINC‑ROR and PRINS for further independent validation.

**Validation of candidate lncRNA by qPCR**

We employed lncRNA specific assays (UCA1, NEAT1, LINC-ROR and PRINS) on a larger cohort of 50 newly diagnosed MM patients, 49 newly diagnosed MGUS patients and 30 HD to test candidate lncRNA expression in exosomal fraction of serum. Results confirmed statistically significant (p ≤ 0.05) difference only in expression of one lncRNA - PRINS - in MM and MGUS patients and HD (Figure 2).

The ROC curve analysis was calculated in order to demonstrate sensitivity and specificity of lncRNA dysregulation. In the validation phase of the study, exosomal PRINS in MM vs. HD had sensitivity of 80.77% (95% CI: 60.6–93.4), specificity of 76.92% (95% CI: 56.4–91.0), AUC = 0.753 with a cut-off value of ≤ ‑0.3676 (Figure 3A). In MGUS vs. HD, sensitivity was 83.33% (95% CI: 62.6–95.3), specificity 80.77% (95% CI: 60.6–93.4), AUC = 0.857 with a cut-off value of ≤ -3.4436 (Figure 3B).

Altogether, statistically significant difference was found when MG (MM and MGUS) patients were compared to HD. MG patients were distinguished from HD with sensitivity of 84.85% (95% CI: 68.1–94.9) and specificity of 83.33% (95% CI: 62.6–95.3), AUC = 0.846 with a cut-off value of ≤ -3.4519.

**Correlation of PRINS expression with biochemical parameters**

In order to determine correlation of lncRNA expression levels with clinical parameters and infiltration of BMPCs, we performed Spearman bivariate correlation. In MM patients, expression levels of exosomal PRINS negatively correlated with BMPCs infiltration (r_s_ = ‑0.422; p < 0.05). In the group of MGUS patients, expression levels of exosomal PRINS negatively correlated with albumin levels (r_s_ = -0.440; p < 0.05) and positively with creatinine levels (r_s_ = 0.512; p < 0.05), β_2_-microglobulin (r_s_ = 0.611; p < 0.005) and lactate dehydrogenase (r_s_ = 0,482; p < 0.05) (Supplementary Table S1).

We did not observe dysregulation of PRINS expression levels between patients at different DS and ISS stages.

**LncRNA expression levels and cytogenetic aberrations association**

Expression levels of PRINS were correlated with typical MM chromosomal aberrations, such as gain(1)(q21), del(13)(q14), del(17)(p13), t(4;14) and hyperdiploidy. Translocation t(4;14) was associated with lower exosomal PRINS levels (p < 0.05) in MM patients (Supplementary Figure S1). No cytogenetics data were available for MGUS patients.

**Analysis of overall survival**

LncRNA expression levels were studied as a possible indicator of survival. Univariate Cox proportional hazards survival model with one explanatory variable showed no significant prognostic impact on OS for exosomal PRINS [HR 0.663 (95% CI: 0.203-2.162), p = 0.496]. Kaplan-Meier analysis was performed; however, no statistically significant relationship of PRINS expression levels with OS was found.

**Discussion**

Expression levels of circulating molecules are potent enough to serve as markers of diagnosis, classification, prognostic assessment of cancer and predictive evaluation of treatment effectiveness (2). For monoclonal gammopathies, including MM, BM biopsies are still the golden standard for diagnosis; these biopsies are unpleasant and sometimes painful for patients. Unlike BM biopsies, liquid biopsies (biopsies of circulating molecules) represent a more available, less painful and more complex approach that can be repeated as often as needed. This could lead to profiling of biologically relevant information for diagnosis as well as monitoring of treatment response and detection of minimal residual disease. For monoclonal gammopathies, liquid biopsies appear to be the way of the future (2). The aim of this two-phase biomarker study was to detect circulating lncRNA molecules in serum of MG patients and HD with possible diagnostic and prognostic potential.

In the first part of the study, only PRINS lncRNA was detected as differentially expressed in MM patients compared to HD (p <0.05). Based on results of profiling, literature and our own results, 4 lncRNA (PRINS, LINC-ROR, NEAT1, UCA1) were selected for validation on a larger cohort of patients. However, in the validation phase, only two lncRNA, UCA1 and PRINS, were detected - and only PRINS remained statistically significant.

PRINS (psoriasis susceptibility-related RNA gene induced by stress) is a lncRNA that has been described in stress-induced psoriasis; its higher expression may increase susceptibility to this disease (13- 15). The gene is located on chromosome 10 (10p12.1); the transcript is about 3.6 kb long. Increased expression of this lncRNA occurs with respect to proliferation and differentiation of keratinocytes and stress factors (UVB, viral infection, translation inhibition). In cells exposed to stress, PRINS has a protective role. Expression of this lncRNA was demonstrated in adrenocortical carcinoma (16). However, expression of PRINS has not been described in hematological malignancies.

In the validation phase of the study, PRINS expression level was significantly different in the exosomal fraction of MM patients compared to HD (p <0.01), and the ROC curves distinguished these groups with sensitivity of 80.77% and specificity of 76.92%. The difference in PRINS expression levels in exosomal fraction of serum was also observed between MGUS patients and HD when the two groups were distinguished with sensitivity of 83.33% and specificity of 80.77%. Overall, statistically significant difference was found when MG (MM and MGUS) patients were compared to HD - with sensitivity of 84.85% and specificity of 83.33% in exosomes.

Routine diagnostic methods reach sensitivity and specificity of 80%. In our study, exosomal PRINS in MM/MGUS vs. HD reached similar values, so these pilot results are comparable to values of standard methods while not requiring invasive BM aspiration. However, at this point, lncRNA cannot be incorporated into the current diagnostic criteria as very limited data concerning these molecules have been published.

When evaluating MM and MGUS patients, PRINS expression was associated with some clinical parameters. Analysis of MM patients showed a negative correlation of PRINS expression in serum exosomes and percentage infiltration of PCs in BM.

Based on our results, it seems that PRINS expression levels are not directly related to MM pathogenesis but may correlate with other processes in the body of the patients.

In MGUS patients, exosomal PRINS expression level negatively correlated with albumin levels, which reflects disease activity. Positive correlations with creatinine, β_2_‑microglobulin and lactate dehydrogenase were observed indicating worse disease prognosis.

Moreover, there was no statistically significant association of PRINS expression and OS in MM patients. Exosomal PRINS expression levels were associated with translocation t(4;14); the presence of this translocation is a negative prognostic factor of MM (1). While the association of PRINS expression with negative prognosis was not confirmed, it may be due to limited availability of cytogenetics data (32/56).

The number of tested lncRNA in this study was limited because of the used Qiagen platform. A more comprehensive platform would have possibly identified more dysregulated lncRNA as candidate molecules for further qPCR verification. In addition, since expression levels of lncRNA in body fluids are lower than levels of lncRNA in cells by its nature, it would be preferable to use a more sensitive method, which would allow detection of unknown lncRNA, i.e., next-generation RNA sequencing.

In our previous study (10), we identified deregulated UCA1 in PCs of MM patients. We included UCA1 in the validation phase of the current study; however, it does not seem to be released into the serum of either group of patients or HD. In addition, in our previous study, we did not find PRINS to be differentially expressed between PCs of MM patients and HD. As we are not sure which tissue the circulating lncRNA are originating from, we did not expect similar results to our previous study.

There is only a limited amount of information available for circulating lncRNA in MM. To the best of our knowledge, three papers have been published so far. In the study of Isin *et al.* (7), they analyzed expression levels of only five chosen candidate lncRNA molecules by qPCR and observed that expression levels of TUG1, MALAT1, HOTAIR and GAS5 were deregulated in MM patients. Correlation of circulating lncRNA with clinical subgroups of MM patients was observed indicating that TUG1 could participate in MM progression. TUG1, MALAT1, HOTAIR and GAS5 were included in the panel of 84 lncRNA tested in our experiment but only TUG1 was detected, but it was not significantly deregulated between MM/MGUS patients and HD. The discordance between our study and study of Isin *et al.* (7) could be based on a different approach (pre-picked candidate lncRNA according to literature, without a screening phase), and more importantly, different type of samples – peripheral blood plasma samples in the case of Isin versus peripheral blood serum in our study.

In the second study, the authors analyzed expression of only one circulating lncRNA- PCAT-1. They showed higher expression of circulating PCAT-1 in MM patients than in HD by qPCR. PCAT-1 was able to distinguish these two groups with 71.7% sensitivity and 93.8% specificity; its levels correlated with serum β_2_-microglobulin levels (19). On contrary, our study used a more comprehensive approach of analysis of 84 lncRNA on a commercial platform and found deregulated expression of PRINS in exosomes of MM and MGUS patients.

The last study published recently showed elevated expression of lncRNA H19 in a cohort of MM patients by qRT-PCR. H19 was shown to correlate with MM staging (20). However, this lncRNA was not expressed in our cohort.

**Conclusion**

Our study indicates a potential of lncRNA as a possible minimally invasive marker of MM and MGUS. However, in order to use lncRNA molecules in the so-called liquid biopsies, further studies are needed.

**Acknowledgements**

The authors would like to thank all the patients and their caregivers for participating in this study. We would like to thank our laboratory technicians from the Department of Clinical Hematology of the Faculty Hospital Brno for technical support. We would like to thank John B. Smith for proofreading the manuscript.

**References**

(1) Rajkumar VS, MD. Updated Diagnostic Criteria and Staging System for Multiple Myeloma. *J Clin Oncol*http://meetinglibrary.asco.org/content/159009-176 (accessed 4 March 2017).

(2) Hocking J, Mithraprabhu S, Kalff A, Spencer A. Liquid biopsies for liquid tumors: emerging potential of circulating free nucleic acid evaluation for the management of hematologic malignancies. *Cancer Biol Med* 2016; 13: 215–225.

(3) Kubaczkova V, Vrabel D, Sedlarikova L, Besse L., Sevcikova S. Cell-free DNA - Minimally invasive marker of hematological malignancies. *Eur J Haematol* 2017; 99: 291–299.

(4) Kubiczkova L, Kryukov F, Slaby O, et al. Circulating serum microRNAs as novel diagnostic and prognostic biomarkers for multiple myeloma and monoclonal gammopathy of undetermined significance. *Haematologica* 2014; 99: 511–518.

(5) Besse L, Sedlarikova L, Kryukov F, et al. Circulating Serum MicroRNA-130a as a Novel Putative Marker of Extramedullary Myeloma. *PloS One* 2015; 10: e0137294.

(6) Manier S, Liu C-J, Avet-Loiseau H, et al. Prognostic role of circulating exosomal miRNAs in multiple myeloma. *Blood*. Epub ahead of print 17 February 2017. DOI: 10.1182/blood-2016-09-742296.

(7) Isin M, Ozgur E, Cetin G, et al. Investigation of circulating lncRNAs in B-cell neoplasms. *Clin Chim Acta Int J Clin Chem* 2014; 431: 255–259.

(8) Derrien T, Johnson R, Bussotti G, et al. The GENCODE v7 catalog of human long noncoding RNAs: analysis of their gene structure, evolution, and expression. *Genome Res* 2012; 22: 1775–1789.

(9) Zhang Q, Su M, Lu G, et al. The complexity of bladder cancer: long noncoding RNAs are on the stage. *Mol Cancer* 2013; 12: 101.

(10) Sedlarikova L, Gromesova B, Kubaczkova V, et al. Deregulated expression of long non-coding RNA UCA1 in multiple myeloma. *Eur J Haematol* 2017; 99: 223–233.

(11) Nemec P, Zemanova Z, Kuglik P, et al. Complex karyotype and translocation t(4;14) define patients with high-risk newly diagnosed multiple myeloma: results of CMG2002 trial. *Leuk Lymphoma* 2012; 53: 920–927.

(12) Durie BGM, Harousseau J-L, Miguel JS, et al. International uniform response criteria for multiple myeloma. *Leukemia* 2006; 20: 1467–1473.

(13) Sonkoly E, Bata-Csorgo Z, Pivarcsi A, et al. Identification and characterization of a novel, psoriasis susceptibility-related noncoding RNA gene, PRINS. *J Biol Chem* 2005; 280: 24159–24167.

(14) Szegedi K, Sonkoly E, Nagy N, et al. The anti-apoptotic protein G1P3 is overexpressed in psoriasis and regulated by the non-coding RNA, PRINS. *Exp Dermatol* 2010; 19: 269–278.

(15) Bari L, Bacsa S, Sonkoly E, et al. Comparison of stress-induced PRINS gene expression in normal human keratinocytes and HaCaT cells. *Arch Dermatol Res* 2011; 303: 745–752.

(16) Glover AR, Zhao JT, Ip JC, et al. Long noncoding RNA profiles of adrenocortical cancer can be used to predict recurrence. *Endocr Relat Cancer* 2015; 22: 99–109.

(17) Mestdagh P, Hartmann N, Baeriswyl L, et al. Evaluation of quantitative miRNA expression platforms in the microRNA quality control (miRQC) study. *Nat Methods* 2014; 11: 809–815.

(18) Wang Y, Barbacioru C, Hyland F, et al. Large scale real-time PCR validation on gene expression measurements from two commercial long-oligonucleotide microarrays. *BMC Genomics* 2006; 7: 59.

(19) Shen X, Zhang Y, Wu X, et al. Upregulated lncRNA-PCAT1 is closely related to clinical diagnosis of multiple myeloma as a predictive biomarker in serum. *Cancer Biomark Sect Dis Markers* 2017; 18: 257–263.

(20) Pan Y, Chen H, Shen X et al. Serum level of long noncoding RNA H19 as a diagnostic
 biomarker of multiple myeloma. *Clin Chim Acta.* 2018 May;480:199-205.

**Figure 1: Expression levels of circulating lncRNA PRINS in screening phase of the study**

Expression levels of specific lncRNA PRINS (p < 0.042) in serum exosomes of 6 MM patients and 6 HD from the screening phase of the study were analyzed using nonparametric Mann-Whitney U test.


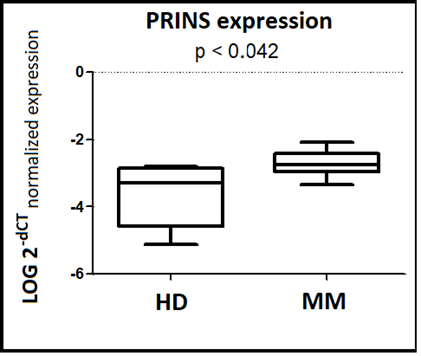


**Figure 2: Circulating lncRNA PRINS in the validation phase of study**

Expression level of lncRNA PRINS in serum exosomes (p < 0.0001) in MM, MGUS patients and HD from the validation phase of the study were analyzed using nonparametric Mann-Whitney U test.


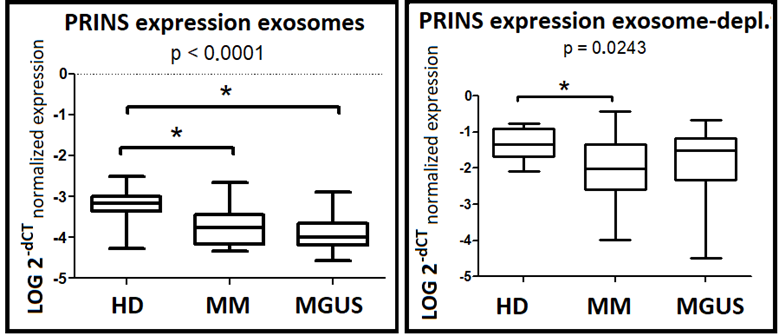


**Figure 3: ROC analysis of lncRNA PRINS in the validation phase of study**

A) serum exosomes in MM patients and HD (sensitivity of 80.77% (95% CI: 60.6-93.4), specificity of 76.92% (95% CI: 56.4-91.0), AUC = 0.753 with a cut-off value of ≤ ‑0.3676)

B) serum exosomes in MGUS and HD (sensitivity was 83.33% (95% CI: 62.6-95.3), specificity 80.77% (95% CI: 60.6-93.4), AUC = 0.857 with a cut-off value of ≤ -3.4436)


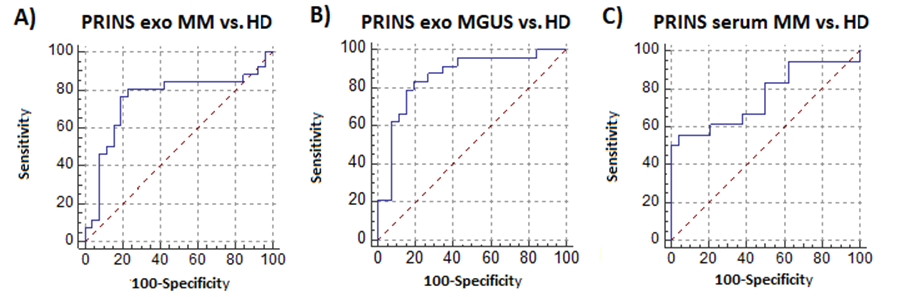


**Supplementary Figure S1: Correlation of translocation t(4;14) with exosomal PRINS levels in MM patients**


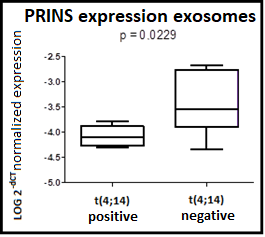


|  | **HD** | **MGUS** | **MM** |
| --- | --- | --- | --- |
| **No. of patients/donors** | 36 | 49 | 56 |
| Gender: males-females | 18-18 | 28-21 | 27-29 |
| Age median (min-max) (years) | 61 (51 - 65) | 66 (35 - 88) | 72 (31 - 89) |
| ISS stage: I-II-III | ND | ND | 14-19-23 |
| D-S stage: I-II-III | ND | ND | 5-9-42 |
| D-S substage: A-B | ND | ND | 38-18 |
| Ig isotype: IgG-IgA-IgM-FLC-Polyclon. | ND | 32-4-10-2-1 | 34-12-0-9-1 |
| Light chains: kappa-lambda | ND | 23-26 | 34-22 |
| **Biochemical parameters: median (min-max)** |  |  |  |
| Hemoglobin (g/L) | ND | 138 (76 - 170) | 98 (62.6 - 157) |
| Thrombocytes (countx109) | ND | 233 (56.5 - 454) | 223.5 (44.8 - 493) |
| Calcium (mmol/L) | ND | 2.4 (2.09 - 2.68) | 2.37 (2 - 4.27) |
| Albumin (g/L) | ND | 43.4 (31.8 - 50.7) | 34.5 (20.8 - 47.7) |
| Creatinine (µmol/L) | ND | 84 (52 - 920) | 93.5 (50 - 923) |
| B2-microglobulin (mg/L) | ND | 2.22 (1.17 - 17.5) | 4.8 (1.7 - 32.7) |
| Lactate dehydrogenase (u kat/L) | ND | 3.7 (1.3 - 6.84) | 3.26 (1.15 - 7.72) |
| C-reactive protein (mg/L) | ND | 1.7 (1 - 40.5) | 5.2 (1 - 111.5) |
| Monoclonal Ig (g/L) | ND | 5.2 (0 - 22.3) | 35.1 (3.3 - 85.6) |
| PCs infiltration of BM (%) | ND | 0.41 (0.1 - 4) | 15 (0.1 - 99.6) |
| **Chromosomal abnormality: positive/negative (%)** |  |  |  |
| 13q14 deletion | ND | 0/6 (12.24%) | 9/23 (57.14%) |
| 17q13 deletion | ND | 2/18 (40.82%) | 22/11 (58.93%) |
| Translocation t(4;14) | ND | 4/14 (36.73%) | 18/16 (60.71%) |
| 1q21 gain | ND | 0/20 (40.82%) | 6/27 (58.93%) |
| Hyperdiploidy | ND | 0/4 (8.16%) | 8/10 (32.14%) |

**Table 1: Baseline characteristics of HD, MM and MGUS patients**

ND‑not defined

**Supplementary Table S1:** **Correlation of serum lncRNA with biochemical parameters**.

For correlation of the data, Spearman coefficient was adopted; significant coefficients of correlation (p < 0.05) are marked with bold and italics.

| **r_s_** | **MM** | **MGUS** |
| --- | --- | --- |
| Hemoglobin (g/l) | -0.096 | -0.091 |
| Thrombocytes (count x 10^9^) | 0.090 | 0.256 |
| Calcium (mmol/l) | 0.221 | -0.123 |
| Albumin (g/l) | -0.310 | ***-0.440*** |
| Creatinine (umol/l) | -0.120 | ***0.512*** |
| β2-microglobulin (mg/l) | -0.058 | ***0.611*** |
| Lactate dehydrogenase (ukat/l) | -0.257 | ***0.482*** |
| C-reactive protein (mg/l) | -0.047 | 0.390 |
| Monoclonal Ig (g/l) | 0.151 | 0.181 |
| PCs infiltration of BM (%) | ***-0.422*** | 0.007 |

|  |  | |  | |
| --- | --- | --- | --- | --- |
|  |  | |  | |
|  |  | |  | |
|  |  | |  | |
|  |  |  | |  |
|  |  |  | |  |
